# Real-Time Visualization of Scent Accumulation Reveals the Frequency of Floral Scent Emissions

**DOI:** 10.3389/fpls.2022.835305

**Published:** 2022-04-18

**Authors:** Hyoungsoo Kim, Gilgu Lee, Junyong Song, Sang-Gyu Kim

**Affiliations:** ^1^Department of Mechanical Engineering, Korea Advanced Institute of Science and Technology, Daejeon, South Korea; ^2^Department of Biological Sciences, Korea Advanced Institute of Science and Technology, Daejeon, South Korea

**Keywords:** floral scent, direct optical measurement, irregular release, floral scent sedimentation, volatile organic component (VOC)

## Abstract

Flowers emit a bouquet of volatiles to attract pollinators or to protect flowers from pathogen and herbivore attacks. Most floral volatiles are synthesized in the cytoplasm of petals and released into the headspace at a specific time of day. Various floral scent sampling methods coupled with gas chromatography-mass spectrometry have been used to measure the quality and quantity of floral volatiles. However, little is known about the emission patterns of floral scents. In most cases, it is still unclear whether floral scents emit continuously or discontinuously. Here we measured the frequency with which lily flowers emit scents using optical interferometry. By analyzing the refractive index difference between volatile organic compounds and ambient air, we were able to visualize the accumulation of the volatile vapors. The frequency of volatile emission was calculated from the unique footprint of temporal power spectrum maps. Based on these real-time measurements, we found that lily flowers emit the volatile compounds discontinuously, with pulses observed around every 10–50 min.

## 1. Introduction

To understand the ecological evolution of plants, the interactions between flowers, pollinators, and enemies—in particular, the frequency of pollinator visitors per flower and per unit time—has been widely explored. Because the fragrance of flowers may affect these interactions (Schiestl, 2010; Farina et al., 2020; Arnold et al., 2021), analyzing and understanding floral scents is crucial.

The term “floral scents” refers to a complex blend of volatile organic compounds [VOCs, Knudsen et al. (2006)]. Most floral scents function to mediate various interactions between plants and animals; some floral scents attract pollen-dispersing mutualists to produce seeds with high genetic diversity (Raguso, 2008), and some scents act as a repellent that functions against particular herbivores (Junker et al., 2011). In addition, a single-scent chemical can act as an attractant and also a repellent in the same plant (Kessler et al., 2019). Floral scents help bees locate other flowers of the same species, which increase the success rate of pollination (Farina et al., 2020; Arnold et al., 2021). To date, about 1,700 floral volatile compounds have been discovered (Knudsen et al., 2006); these compounds are largely classified as terpenoids, phenylpropanoids, and fatty acid-derivatives according to their biosynthetic pathways (Knudsen et al., 2006; Knudsen and Gershenzon, 2020). The timing of floral scent emission is also important for pollination success. Many flowering plants are able to synchronize their scent emission rhythms with times when pollinators are active for pollination. For instance, flowers in *Petunia* *axillaris* (Oyama-Okubo et al., 2005) and the wild tobacco, *Nicotianaattenuata* (Kessler et al., 2008; Yon et al., 2016) emit volatile compounds at night to attract a night-active pollinator (Fenske et al., 2015; Yon et al., 2016). By characterizing floral scents and emission patterns, we can shed light on the ecological interactions between flowers and pollinators.

To measure floral scent emissions, adsorbent media is exposed to the headspace above flowers for a certain period of time. Headspace solid-phase extraction coupled to gas chromatography mass spectrometry (GC-MS) has been used to identify the scent compounds and to measure the quantity of each compound (Tholl et al., 2006; Burkle and Runyon, 2017). However, because the headspace volatiles are normally collected by time unit (hourly), whether floral scents are emitted continuously or discontinuously has been ignored. Recently, several analytical systems have been developed to monitor volatile emissions with high temporal resolution. A portable GC, e.g., zNose^TM^ and Torion T-9, separates and analyzes volatile compounds within several minutes after a short sampling period (Tholl et al., 2006). The proton transfer reaction-mass spectrometry (PTR-MS) can perform real-time measurements of flower volatiles by directly injecting volatiles into a PTR-MS (Powers et al., 2020). A laser-based gas detection system monitors light intensity or frequency absorbed by volatile compounds with continuous airflow (Harren and Cristescu, 2013). Furthermore, a smartphone-based VOC-detection system using chemo-responsive dyes has been introduced (Li et al., 2019). Although these instruments allow researchers to monitor the emission of floral volatiles in real-time or near-real-time, access to the instruments is limited due to their price and complexity. Furthermore, these instruments have been used to monitor real-time volatile emissions from plants exposed to abiotic and abiotic stress. As a result, their ability to measure the emission frequency of floral VOCs in an ambient environment, which is crucial to understand the emission mechanism of floral scents, is limited. To solve this issue, we suggest using an optical measurement technique to detect VOCs in the air, as summarized in Table 1. If we can detect the refractive index difference depending on the concentration of the gas media (i.e., VOCs), we should be able to measure temporal and spatial signals from flowers. Here, we develop and use the Mach-Zehnder interferometry system to monitor floral volatile emission in the lily. Using this instrument, we ask whether lily flowers emit scent compounds continuously or discontinuously.

**Table 1:** Current gas sensing measurement techniques (◌: Good, △: Fair, × : Bad).

	**Sensing mechanism**	**Sensitivity**	**Real time measurement**	**Spatial measurement**
Electrochemical sensor (Tierney and Kim, 1993; Bhoga and Singh, 2007; Dossi et al., 2012)	Electromotive force	◌	◌	×
Catalytic combustion sensor (Han et al., 2007; Kim and Lee, 2014)	Electrical conductivity	×	◌	×
Semiconductor sensor (Dey, 2018)	Electrical conductivity	×	◌	×
Laser-based gas sensor (Richter et al., 2000; Werle et al., 2002)	Wavelength absorption rate	△	◌	×
Gas chromatography-mass spectrometer (Karasek and Clement, 2012)	Chemical affinity and mass	◌	×	×
Proton transfer reaction-mass spectrometer (Hansel et al., 1995; de Gouw and Warneke, 2007)	Proton affinity and mass	◌	×	×
Chromatography (McNair et al., 2019)	Chemical affinity	◌	×	×
Optical interferometry (Toker and Stricker, 1996; Dehaeck et al., 2014)	Refractive index	△	◌	◌

## 2. Results and Discussion

Using a laser interferometer, we investigated how often a flower emits a fragrance. The interferometer is able to detect the release of volatile organic components by measuring the relative refractive index (Δ*n*) between VOCs and air in time and space. We believe that our method can be used to explore dynamic interactions between plants and insects.

### 2.1. Real-Time Measurement of the Emission of Floral Scents

**Figure 1:**
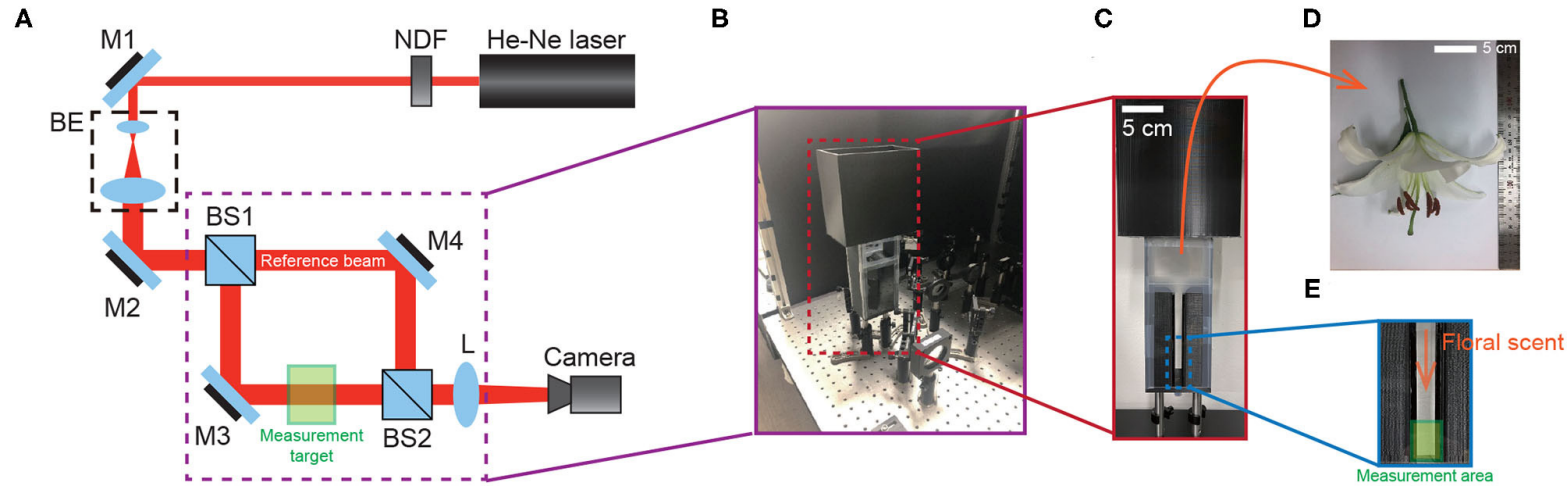
Experimental setup. **(A)** Schematic of Mach-Zehnder interferometer. Red lines indicate the optical ray from He-Ne laser. NDF, neutral density filter; BE, beam expander; BS, beam splitter; M, Mirror; L, achromatic lens (f = 150 mm). Measurement target contains a flower during the measurement of floral scent. **(B)** Illustration for the setup for interference between a reference beam and a measurement target **(C)** Flower container to collect the floral scent from lily. **(D)** An actual flower. **(E)** The 2D narrow channel for the measurement area.

**Figure 2:**
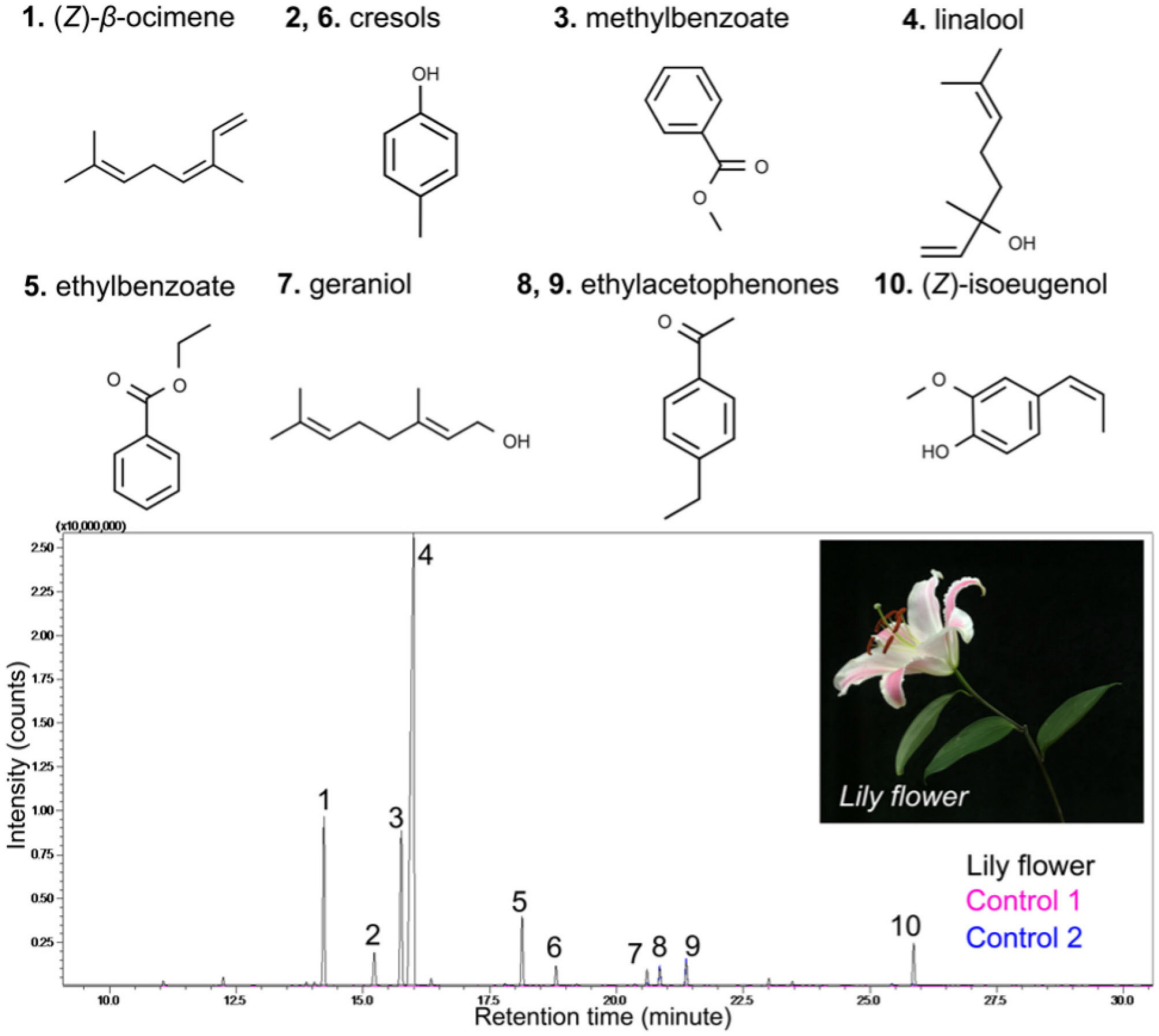
Gas chromatograms of lily floral volatiles. Floral headspace volatiles were collected from one lily flower. The major peaks (compounds) were identified by comparing mass spectra with spectra of a NIST library. Labeled compounds are: (1) (Z)-β-ocimene, (2) cresol, (3) methylbenzoate, (4) linalool, (5) ethylbenzoate, (6) cresol, (7) geraniol, (8, 9) ethylacetophenones, and (Z)-isoeugenol. Control 1 and 2 show any ambient contaminants in the air.

To directly see the signal of the floral scent from the lily flower, we set up the Mach-Zehnder interferometry with a closed hood box for lily, as shown in Figure 1. Using GC-MS to measure the components of VOCs from the lily, we had already found that linalool is one of the main components (see Figure 2). In this case, *n*_*linalool*_ = 1.463 and *n*_*air*_ = 1.000293 (Linstorm, 1998). The molar mass of linalool (C_10_H_18_O) is 154.253 g/mol, which is much heavier than that of an ambient gas, typically 28.97 g/mol at room temperature (T = 295 K). So, we presumed that if linalool is released from the lily, the molecules will sediment to the downward due to gravity. See more details about the sedimentation of linalool's vapors in the Methods. Based on this, we hung the lily upside down and designed the measurement container in Figure 1B. The lily's VOCs of lily—here mostly linalool—settle along a narrow channel and accumulate at the bottom, where they are captured by the Mach-Zehnder interferometry.

We recorded the interference signal of the lily's VOCs at the measurement area (i.e., the greenish box in Figure 1. As long as the fragrance molecules accumulate at the bottom of the 2D channel (see Figures 1C,E), the phase of the fringe pattern will be shifted to one side, which is due to the refractive index change by the VOCs in the box. While the VOCs invaded and replaced the ambient gas, we measured the images every 10 min, the maximum recording time for the DSLR camera (Nikon 5,000, Japan). After each experiment, we cleaned up the flower container and reset the experiments for 20 min. Based on the Mach-Zehnder experiments, we measured the time-dependent phase shift in 2D, ϕ(*x*, *y*, *t*). Here, ϕ(*x*, *y*, *t*) is related to the vapor concentration from the lily. Because the principle of the measurement technique is to detect and compare the refractive index change by the concentration of vapors, the mole fraction of VOCs of lily is proportional to the magnitude of the gradient of ϕ (*x, y*).

**Figure 3:**
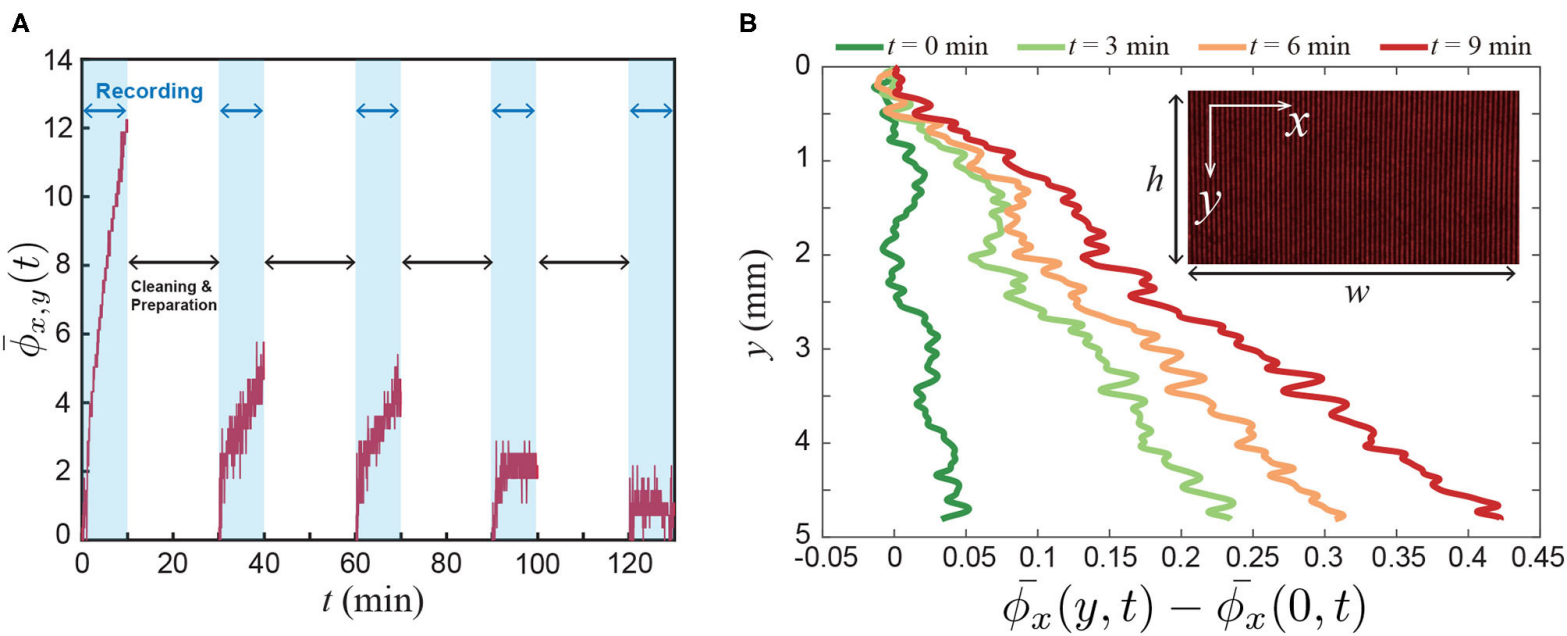
Experimental results. **(A)** Spatially averaged phase signal ${\bar{\phi }}_{x,y}\left(t\right)$ from the Mach-Zehnder interferometry, where ${\bar{\phi }}_{x,y}\left(t\right)$ = $\frac{1}{wh}\int_{0}^{h}\int_{0}^{w}\phi \left(x,y,t\right)$d*x*d*y*, and *w* and *h* are the horizontal and vertical lengths of the field of view, respectively. Here, ϕ(*x, y, t*) is related to the mole fraction of the floral scent of a lily. The signal was recorded for 10 mins using a DSLR camera. After then, the flower container was cleaned during the data transfer from the camera to a computer. We performed the sequential 5 experiments with the same lily. During the experiments, water was provided through the small vial at the tip of the stem. **(B)** Local signal of the floral scent along the vertical direction. ${\bar{\phi }}_{x}\left(y,t\right)$ = $\frac{1}{w}\int_{0}^{w}\phi \left(x,y,t\right)$d*x*.

Figure 3A shows that the spatially-averaged phase signal changes over time, ${\bar{\phi }}_{x,y}\left(t\right)$ = $\frac{1}{wh}\int_{0}^{h}\int_{0}^{w}\phi \left(x,y,t\right)$d*x*d*y* where *w* = 8 mm and *h* = 5 mm are the horizontal and vertical lengths of the field of view, respectively. From this result, we can confirm that the vapor molecules are released from the flower and those accumulated from the bottom. Furthermore, to examine the local accumulation of the floral scent along the vertical direction, we averaged the phase signal along the *x*-direction and plotted the results in Figure 3B. Then, we observed that the refractive index changed over time, which shows the non-uniform vapor distribution in the box along the *y*-direction. Also, the flux of the floral scent is not constant because [${\bar{\phi }}_{x}\left(4,t\right)-{\bar{\phi }}_{x}\left(0,t\right)$] varies over time (see Figure 3B).

**Figure 4:**
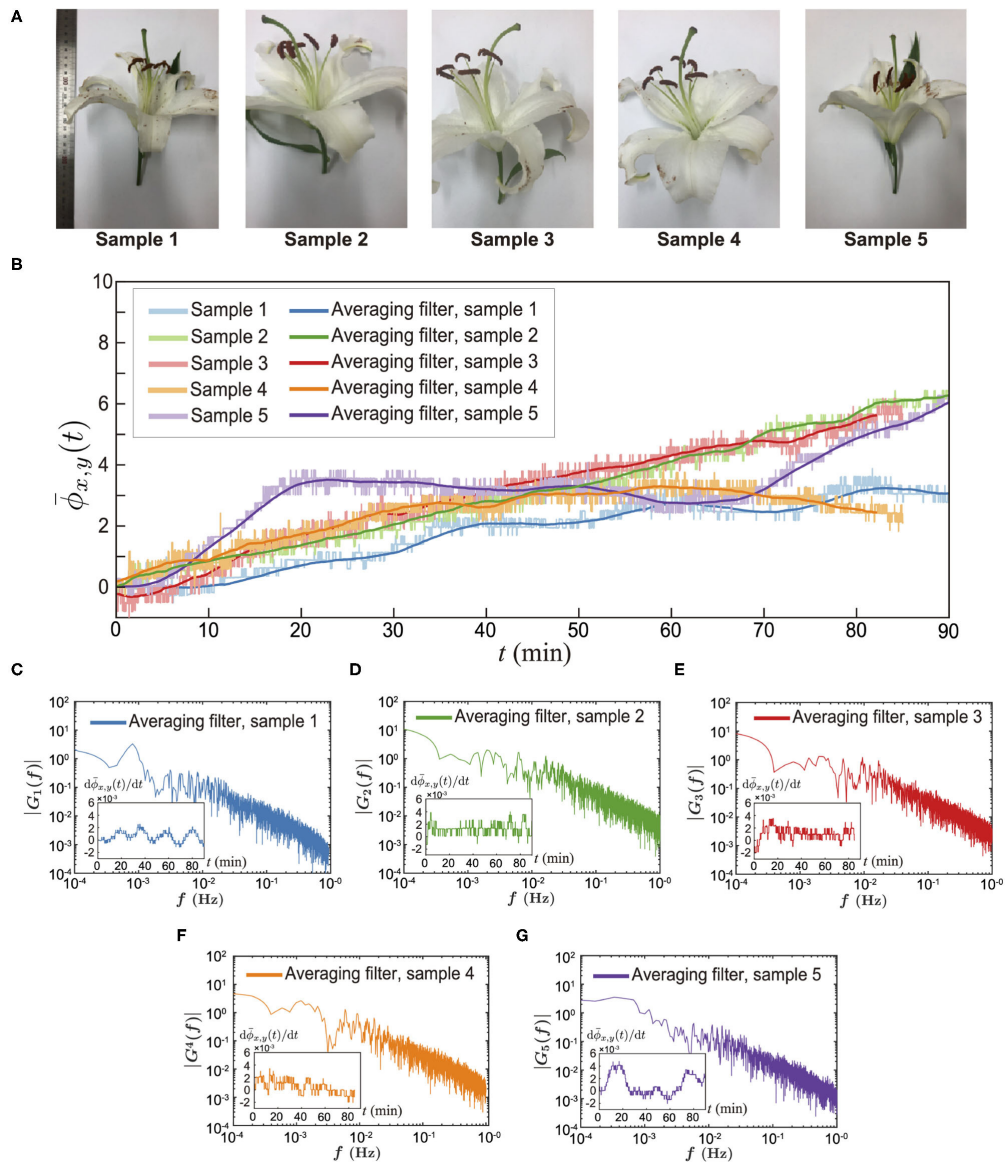
Direct measurement of floral scents from alive lily. **(A)** Sample pictures. **(B)** Spatially averaged phase signal for 90 mins. The pastel colors are ${\bar{\phi }}_{x,y}\left(t\right)$ for different samples. The dark colors represent the averaged data using a spatial filter. **(C–G)** Amplitude spectrum results of the time-dependent net flux of the floral scent emission [d${\bar{\phi }}_{x,y}$(*t*)/d*t*], which were obtained from Fast Fourier Transforms (MATLAB subroutine FFT). The inset of each figure is a raw data of the time-dependent floral scent emission rate, d${\bar{\phi }}_{x,y}$(*t*)/d*t*.

For the continuous and long-duration experiments, we used a USB camera (Ximea) that can record for over 1 h (see Methods). Because it has a relatively low spatial resolution compared to the DSLR camera, we mainly investigated a spatially-averaged phase signal for the whole measurement area (*h* × *w*). Finally, we performed long-duration experiments for several flowers, as shown in Figure 4. We randomly selected 5 different lilies (see Figure 4A) and executed the experiments on a different date but during the same time slot. We conducted experiments for 90 min beginning at noon under the same experimental conditions (see detailed conditions in Methods). We observed that the spatially-averaged phase signals increased over time for all cases (see Figure 4B). The pastel colors indicate the real-time measurement results and the dark color of each pastel color represents the mean profile that is obtained from a moving average filter. We observed that the profiles do not have a constant slope. However, ${\bar{\phi }}_{x,y}\left(t\right)$ monotonically increased with different fluctuations.

To examine the flux of the lily's scent, we calculated $\frac{d}{dt}{\bar{\phi }}_{x,y}\left(t\right)$. Using these data, we performed a fast Fourier transform (FFT) to measure the frequency of the lily's scent flux, as shown in Figures 4C–G. The insets are raw profiles of $\frac{d}{dt}{\bar{\phi }}_{x,y}\left(t\right)$ for each lily. Although the signal contains some noise, we could see a rhythmical trend from 5 randomly chosen lilies. The frequencies of the flux of the floral scent are presented in Figures 4C–G. The first sample showed a 0.81 mHz lasting about 21 min. The second, third, fourth, and fifth samples presented 8, 8, 14, and 51 min, respectively. With the current measurement technique, we measured the emission rate of the lily's floral scent. Although various reports in literature have suggested that the visiting frequency of insects could be correlated with VOCs emission rates, it is still far from complete to confirm the relation between the unsteady visiting trend of insects and the floral scent emission rates. However, we believe that the current direct measurement results could be a key result to bare out this assumption.

**Figure 5:**
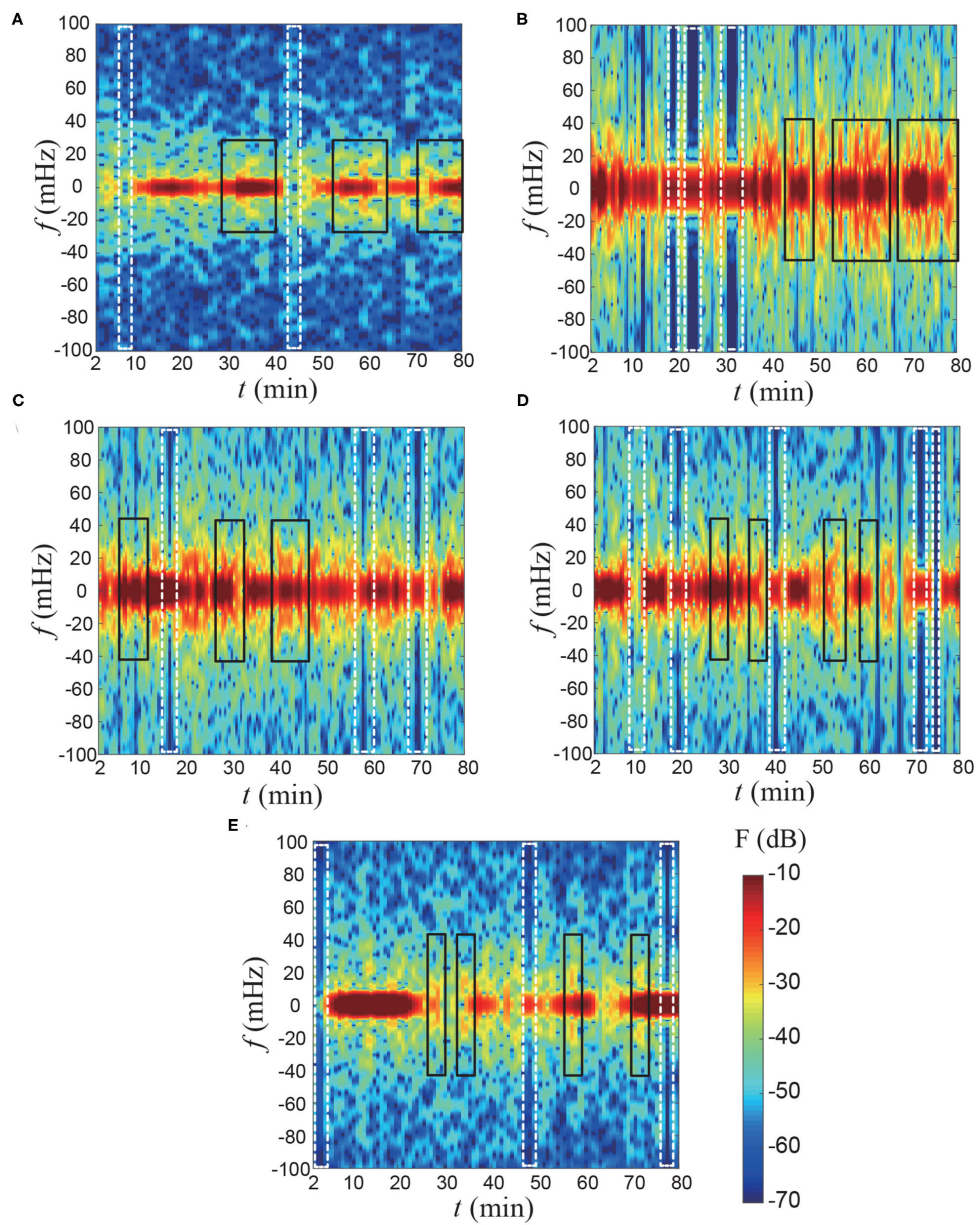
Temporal power spectrum results of the flux of floral scents. The results are reconstructed based on Figure 4B using a moving average filter for every 5 min. **(A)** Sample 1, **(B)** Sample 2, **(C)** Sample 3, **(D)** Sample 4, and **(E)** Sample 5. The contours represent a magnitude **(F)** of the power spectrum. The white dashed-box indicates that the fragrance is temporally stopped emitting. The black rectangular box highlights the similar temporal power spectrum signal for each flower.

Additionally, based on the Mach-Zehnder interferometer results, we can also obtain temporal power spectrum results where the contours represent a magnitude (F) of the power spectrum. Figure 5 showed the unsteadiness of the temporal power spectrum signals. The results indicate that over a short time period, the lily irregularly releases floral scents in both low- and high-frequency domains. First, the VOCs' emission signals that are highlighted in the white dashed-box (Figure 5) temporally disappear, suggesting that the lily either stops or slows down their release. Second, each flower might have a specific footprint of temporal power spectra. There are somewhat similar signals that are marked as a black rectangular box. The structure of the footprint images are not identical when the flower is different. We think that the individual emission patterns could be related to various factors including genotypes and horticultural environments. To be honest, at this moment, it is difficult to say what could be the reason. However, at least, we showed for the first time the pulsed emission of volatile organic components.

## 3. Conclusions and Outlook

In this study, we observed that a lily discontinuously releases fragrances over time. The emission of VOCs was visualized by detecting the relative refractive index of linalool emitted by a lily. The temporal power spectrum indicates that each lily has its own profile of floral scents. The emission frequency of each flower might differ according to genotypes and/or growth conditions. However, the rate of emission is less than 1 h in all the lilies. Despite the ecological importance of floral volatiles, how flowers emit scent compounds remains unknown. Recently, Natalia Dudareva and colleagues demonstrated that floral volatiles in *Petuniahybrids* are actively transported across a plasma membrane from cytosol to cell wall by an adenosine triphosphate-binding cassette (ABC) transporter (Adebesin et al., 2017). Protein synthesis, localization, and the activity of the ABC transporter would affect discontinuous release of lily's floral scents. The floral scents passing through the ABC transporter likely move through the cell wall and lipophilic cuticle layer (Liao et al., 2021). While the transport mechanism of the cuticle remains unknown, Liao et al. (2021) found that the 50% of internal floral scents were detected in the cuticle layer of petunia flowers. Scent compounds might pass through the cuticle layer when a certain level of chemicals accumulates, explaining the discontinuous rhythm of their emission. To unpuzzle the reason of the irregular floral scent emission, we think it needs to connect to biology much more strongly. Although we could not verify this point at this moment, we believe that the current measurement results can provide a new insight to understand and to further explore the biosynthesis and emission mechanism of floral volatiles.

## 4. Methods

### 4.1. Plant Materials and Volatile Analysis

Lily flowers (*Lilium* Oriental Hybrid “Siberia”) were used in this study. Cut flowers were obtained from local growers and transferred to our growth room (16 h light/8 h dark conditions at 26°C with ± 2°C variation). To collect floral volatiles, one fully opened flowers were put in a 7 L glass chamber. Headspace volatiles were trapped in silicone tubing (polydimethylsiloxane, PDMS) and analyzed as previously described in Kallenbach et al. (2014). Briefly, a clean PDMS tube (1 cm in length) was placed directly over the lily flower for 1 h, and GCMS-QP2020 (Shimadzu, Kyoto, Japan) coupled with a thermal desorption unit (TD-30, Shimadzu) containing the PDMS tube was used for volatile analysis. Volatiles were separated on an Rtx-5MS capillary column (30 m × 0.25 mm; 0.25 μm; Shimadzu) using helium as a carrier gas. The GC-MS program was operated in split mode at a 1/20 ratio (250°C), with the oven temperature starting at 40°C for 5 min, increasing by 5°C min^−1^ to 185°C, and by 30°C min^−1^ to 280°C and holding for 0.83 min. See the results in Figure 2. Volatile compounds were identified by comparison of spectra against a library (NIST database), and, where possible, by comparison to standards. Chemical standard compounds (Ocimene, W353901; linalool, W263516) used in this study were purchased from Sigma-Aldrich (USA).

### 4.2. Experimental Condition and Setup for Optical Interferometry

During the experiment, water was provided through the small vial at the tip of the stem of the lily. We kept the experimental conditions that the temperature was 295 ± 1 K and the relative humidity was 45 ± 6 %. The CO_2_ concentration was kept as 588 ± 140 ppm in the lab all the time.

To measure the signal of the volatile organic compounds released from lilies, we used the Mach-Zehnder interferometry experimental setup (see illustration in Figure 1). The 632 nm He-Ne laser diode (10 mW, Melles Griot 25-LHP-991) was used as the light source. The intensity of the laser was adjusted using the metallic neutral density filter (NDF, Thorlabs Inc. NDL-25S-4) and the beam size of the light is expanded through the beam expander (BE) to observe the accumulation of the organic vapors in the chamber. The single laser source is precisely split into 50:50 % at the first beam splitter (BS1), and then the sample beam and the reference beam were interfered each other at the second beam splitter (BS2). The detailed experimental technique is summarized in the preceding study (Lee and Kim, 2018). The interference patterns were captured by cameras (Nikon D6500 having 1920 × 1080 pixel and Ximea camera having 1280 × 1024 pixel) whose the nominal sizes of the image sensors are 23.5 × 15.7 and 6.2 × 5 mm, respectively. The recording frame rate is 60 frames per second for DSLR and 30 frames per second for Ximea. The spatial frequency of the resulting interference pattern in measurements were set to about 11–14 mm^−1^. The DSLR camera has a spatial resolution 5.3 μm/pixel and the Ximea camera has 4.9 μm/pixel. In this study, we reported 5 representative results for the emission rate of VOCs of lily among several experiments.

### 4.3. Post-processing for Fringe Pattern Results

The interference fringe pattern obtained from the Mach-Zehnder interferometer was described as


(1) $$\begin{array}{r} g\left(x,y\right)=a\left(x,y\right)+b\left(x,y\right)\text{cos}\left[2\pi f_{x,c}x+\phi \left(x,y\right)\right], \end{array}$$

where *a*(*x, y*) is a background, *b*(*x, y*) is a fringe modulation, and *f*_*x, c*_ is a spatial frequency. ϕ(*x, y*) is represented as volatile component concentration from lily (see the inset of Figure 3B).

To extract ϕ(*x, y*) from Equation (1), we used 2D Fourier transform proilometry method (Takeda and Mutoh, 1983; Lin and Su, 1995). Using Euler's formula, the complex form of Equation (1) is


(2) $$\begin{array}{r} g\left(x,y\right)=a\left(x,y\right)+c\left(x,y\right)e^{j2\pi f_{x,c}x}+c^{*}\left(x,y\right)e^{-j2\pi f_{x,c}x}, \end{array}$$

where $c\left(x,y\right)=\frac{1}{2}e^{j\phi \left(x,y\right)}$ and $c^{*}\left(x,y\right)=\frac{1}{2}e^{-j\phi \left(x,y\right)}$ are complex conjugate. Equation (2) is 2D Fourier transformed with respect to *x* and *y* as


(3) $$\begin{array}{r} G\left(f_{x},f_{y}\right)=A\left(f_{x},f_{y}\right)+C\left(f_{x}-f_{x,c},f_{y}\right)+C^{*}\left(f_{x}+f_{x,c},f_{y}\right), \end{array}$$

where $G\left(f_{x},f_{y}\right)=F\left[g\left(x,y\right)\right]$, $A\left(f_{x},f_{y}\right)=F\left[a\left(x,y\right)\right]$, $C\left(f_{x}-f_{x,c},f_{y}\right)=F\left[c\left(x,y\right)e^{j2\pi f_{x,c}x}\right]$, $C(f_{x}+f_{x,c},f_{y})=F[c{\left(x,y\right)}^{*}e^{-j2\pi f_{x,c}\;x}]$, respectively. F is a Fourier operator. The spatial carrier frequency *f*_*x, c*_ was obtained from the reference fringe pattern without the droplet. By using this information, *C*(*f*_*x*_−*f*_*x, c*_, *f*_*y*_) can be shifted to *C*(*f*_*x*_, *f*_*y*_), which is 2D inverse Fourier transformed as *c*(*x, y*). Finally, the phase difference ϕ(*x, y*) can be obtained by the relation ϕ(*x, y*) = Im[ln[*c*(*x, y*)]]. The measured phase results ϕ_*s*_(*x, y*) reconstructed using fast cosine transforms and least-squares method. Then, to make the distribution of phase continuous, we used the phase unwrapping algorithm by Ghiglia and Romero (1994).

To obtain the temporal power spectrum, we used the function stft (Short-Time Fourier Transform) of MATLAB 2020a with a Kaiser window of length 512 data points and shape parameter 5. The scale of the temporal window is approximately 2–5 min with an overlap ratio 78%. All the data set were segmented for about 30–66 s.

### 4.4. Sedimentation of Volatile Organic Components

Here, the relative atomic mass of evaporated vapors in air, so Δ*u* = (*M*_*u*_ of linalool - *M*_*u*_ of air)/*N*_*A*_ > 0, where *N*_*A*_ is the Avogadro's number, 6.0221415 × 10^23^ mol^−1^. To further estimate the gravitational effect, a dimensionless parameter, Rayleigh number (Ra), can be considered, which is the ratio between gravitational effect and viscous and diffusion effects (Dietrich et al., 2003). Ra number can be defined as Δ*ρgw*^3^/μ*D*, where *g* (gravitational acceleration) = 10 m/s^2^, *w* (channel width) = 8 mm, μ (viscosity of air) = 10^−5^ Pa·s, *D* is the diffusion coefficient of linalool in air. Here, Δρ (= ρ_*linalool*_ - ρ_*air*_) = 4.3 g/cm^3^ (Linstorm, 1998). In general, the diffusion coefficient of the alcohol component ranges from *O*(10^−6^ m^2^/s) and *O*(10^−5^ m^2^/s). Then, we can estimate Ra ≫ 1, so that the emitted linalool vapors can be sedimented along the channel.

## 5. Data Availability Statement

The original contributions presented in the study are included in the article/supplementary material, further inquiries can be directed to the corresponding authors.

## Author Contributions

HK and S-GK initiated and conceived the project and planned the experiments and wrote the first draft of the manuscript. HK and GL carried out the experiments. GL conducted data processing. JS and S-GK performed the biological experiments. HK, GL, JS, and S-GK analyzed the data. HK and GL contributed equally to this work. All authors discussed and edited the manuscript.

## Funding

This work was based on research which has been conducted as the KAIST-funded Global Singularity Research PREP-Program. HK was partially supported by Basic Science Research Program through the National Research Foundation (NRF) of Korea funded by the Ministry of Science (NRF- 2021R1A2C2007835). S-GK was partially supported from the Rural Development Administration (PJ016403) in South Korea.

## Conflict of Interest

The authors declare that the research was conducted in the absence of any commercial or financial relationships that could be construed as a potential conflict of interest.

## Publisher's Note

All claims expressed in this article are solely those of the authors and do not necessarily represent those of their affiliated organizations, or those of the publisher, the editors and the reviewers. Any product that may be evaluated in this article, or claim that may be made by its manufacturer, is not guaranteed or endorsed by the publisher.
